# Multimodal Detection of Agitation in People With Dementia in Clinical Settings: Observational Pilot Study

**DOI:** 10.2196/68156

**Published:** 2025-07-15

**Authors:** Abeer Badawi, Somayya Elmoghazy, Samira Choudhury, Sara Elgazzar, Khalid Elgazzar, Amer M Burhan

**Affiliations:** 1 Internet of Things (IoT) Research Lab, Department of Electrical and Computer Engineering, Ontario Tech University, 2000 Simcoe Street North, Oshawa, ON, L1G 0C5, Canada, 1-905-721-8668 ext. 7365; 2 Ontario Shores Centre for Mental Health Sciences, Whitby, ON, Canada; 3 Department of Psychiatry, Western University, London, ON, Canada; 4 Faculty of Science, Ontario Tech University, Oshawa, ON, Canada; 5 Canadian University of Dubai, Dubai, United Arab Emirates; 6 Temerty Faculty of Medicine, University of Toronto, Toronto, Canada

**Keywords:** dementia, agitation, artificial intelligence, wearable sensors, video detection, multimodal sensing

## Abstract

**Background:**

Dementia is a progressive neurodegenerative condition that affects millions worldwide, often accompanied by agitation and aggression (AA), which contribute to patient distress and increased health care burden. Existing assessment methods for AA rely heavily on caregiver reporting, introducing subjectivity and inconsistency.

**Objective:**

This study proposes a novel, multimodal system for predicting AA episodes in individuals with severe dementia, integrating wearable sensor data and privacy-preserving video analytics.

**Methods:**

A pilot study involving 10 participants was conducted at Ontario Shores Mental Health Institute. The system combines digital biomarkers collected from the EmbracePlus (Empatica Inc) wristband with video-based behavioral monitoring. Facial features in video frames were anonymized using a masking tool, and a deep learning model was used for AA detection. To determine optimal performance, various machine learning and deep learning models were evaluated for both wearable and video data streams.

**Results:**

The Extra Trees model achieved up to 99% accuracy for personalized wristband data, while the multilayer perceptron model performed best in general models with 98% accuracy. For video analysis, the gated recurrent unit model achieved 95% accuracy and 99% area under the curve, and the long short-term memory model demonstrated superior response time for real-time use. Importantly, the system predicted AA episodes at least 6 minutes in advance in all participants based on wearable data.

**Conclusions:**

The findings demonstrate the system’s potential to autonomously and accurately detect and predict AA events in real-time. This approach represents a significant advancement in the proactive management of behavioral symptoms in dementia care.

## Introduction

### Background

Dementia is a neurodegenerative condition that leads to a progressive decline in cognition and is one of the leading causes of death, disability, and hospitalization in Canada and worldwide. Currently, dementia is the seventh cause of death worldwide [1]. Worldwide, over 55 million individuals are living with dementia; as the ratio of older people increases, this number will grow to 78 million by 2030 and 139 million by 2050, making dementia a major global health crisis [1]. In addition to cognitive and functional decline, people living with dementia also experience noncognitive neuropsychiatric symptoms (NPS) during their illness [2]. NPS commonly includes agitation, aggression, apathy, symptoms of psychosis, delusions, hallucinations, and disturbances of sleep and appetite. Among NPS, agitation and aggression (AA) occur frequently in severe cases and are a common source of distress for patients and caregivers [3]. They commonly occur during care and are believed to be manifestations of perceived or real unmet needs [3]. Behaviors of AA include pacing, rocking, gesturing, restlessness, shouting, throwing objects, and destroying property [4]. These symptoms are the leading cause of hospitalizations, extended length of stay of inpatients, and increased demand for placement in long-term care facilities [5]. AA enormously burdens people living with dementia, their families, caregivers, and health care systems.

In current practices, AA is commonly assessed through caregiver reports. Many observational methods have been developed, including the Neuropsychiatric Inventory [6] and the Cohen-Mansfield Agitation Inventory [7]. These assessments are based on manual observations, which are subject to potential bias depending on the caregiver’s memory or emotional state. It is possible to address these limitations by using artificial intelligence (AI) and predictive algorithms to predict episodes of AA in people living with dementia. In 2025, AI technologies are expected to be worth an estimated US $36 billion [8]. There is growing evidence that combining AI and sensory technologies to develop a solution for NPS detection will guide the provision of personalized interventions for people living with dementia [9-11]. The timely detection of critical events in people living with dementia using digital technologies is gaining wide acceptance [12,13]. Predicting and managing AA in people living with dementia requires innovative approaches that integrate multiple data sources. Wearable sensors and video-based monitoring systems provide unique opportunities for the real-time detection of AA and preagitation behaviors, but challenges such as privacy concerns and scalability have limited their adoption in clinical settings. This study addresses these challenges by combining biometric data from wearable sensors with AI-driven video analysis, enabling real-time detection and prediction of AA. This integrated approach aims to facilitate timely interventions, reduce care costs, and improve outcomes for both patients and caregivers. We conducted a pilot study in the Geriatric Dementia Unit (GDU) and the Geriatric Transitional Unit at the Ontario Shores Center for Mental Health Sciences [14]. We combine biometric data from the EmbracePlus wristband [15] and video data from CCTV cameras installed in common areas in both units. Machine learning and deep learning techniques, including Extra Trees, Gradient Boosting, Random Forest, multilayer perceptron (MLP), and recurrent neural networks (RNNs), are used to analyze biometric and skeletal data extracted from both the wristband and the video cameras. We achieved high accuracy in detecting AA from the EmbracePlus wristband through comprehensive data preprocessing, feature extraction, the Extra Trees classification algorithm for personalized models, and the MLP algorithm for the general model. Additionally, AA detection was enhanced by analyzing real-time video feeds with skeletal key points and using RNN-based neural networks, particularly long short-term memory (LSTM) and gated recurrent unit (GRU) [16]. These networks, optimized for real-time processing, facilitate timely interventions. The pilot study demonstrated the system’s effectiveness through both the wristband and video detection.

### Related Work

The growing number of people living with dementia causes significant challenges for health care systems and caregivers. One of these challenges is to deal with symptoms of AA that increase with the severity of dementia. Recent advancements in multimodal sensing technologies, including cameras and wearable wristbands, have shown promise in monitoring and managing AA in people living with dementia [10,17,18]. Wearable devices, capable of capturing physiological signals such as acceleration, heart rate, and skin conductance, have demonstrated potential for real-time AA detection [19-21]. Recent advancements in wearable sensor technology and AI have shown promise in addressing the early detection of AA behavior when focusing on signal processing and machine learning to extract features and classify AA events [22].

However, real-time video-based monitoring systems to monitor AA behavior in dementia patients are an area of interest for researchers today [10,17,23]. These systems use cameras positioned in patients’ rooms or common areas to consistently record and monitor patients’ behavioral data. Some research [24,25] used video cameras to detect AA from previously recorded videos at the Specialized Dementia Unit, Toronto Rehabilitation Institute, Canada. Their system focused on offline AA detection. Another work collected the training dataset from healthy people who imitated agitated hand movements [26]. Researchers have highlighted the importance of real-time feedback, which allows for timely interventions and reduces the severity and duration of AA events. This can help improve the quality of life and reduce the stress caused by agitated behaviors in both patients and caregivers. Researchers have also considered privacy factors and concerns while ensuring the accuracy of these systems. The work done in studies by Mishra et al [24] and Marshall [26] has proposed different masking methods for the people in the video frames that allow for AA detection while keeping the patients’ identities and features hidden.

Multimodal sensing, which combines data from multiple sensors, types, and sources, has emerged as a promising approach to understanding AA in people living with dementia [27,28]. It enhances the detection of early signs of AA and the identification of relevant triggers [23,29]. Clinical trials evaluating the use of multimodal sensing in the context of dementia and AA behavior are crucial in this type of research. These trials assess the feasibility and efficacy of monitoring systems that combine various data sources to inform clinical decision-making. The results of these trials will provide valuable insights into the practical implications of multimodal sensing in real-world health care settings. Most existing studies on video systems and wearable sensors for AA detection in real-time have been conducted in controlled laboratory settings or residential care facilities with limited datasets [18,30]. Research is needed to use more diverse and general datasets gathered and applied in real hospital settings. This is essential since the data from hospitals would be more representative of people with severe dementia who are more prone to AA. Research in such a real-world setting with data gathered from real patients is vital for developing realistic and applicable solutions and ensuring effective treatment and care for people living with dementia [18,30].

Moreover, it is challenging to identify the actual start and end times of AA episodes. This is because the AA events are extracted from nurse notes, which are not accurate and prone to human or individual error. Many AA episodes may also be overlooked and mislabeled as nonagitation. In addition to the datasets, there is a high demand for accurate and reliable end-to-end real-time monitoring solutions to actively predict and respond to AA events. There is also still a need for an in-depth investigation of AI and its features in addition to an in-depth investigation of preagitation patterns and signs that could trigger AA in people living with dementia. These investigations are necessary to indicate the usefulness of preagitation signs and patterns in early prediction. Digital biomarkers can help detect and predict AA early in real time [31]. The investigation by Alam et al [32] shows the correlation between motion biomarkers collected from the accelerometer and the early AA signs, which is particularly useful for personalized AA detection models.

This paper presents a unique system that combines video analysis and wearable sensor data to predict AA in people living with dementia. The video feeds are crucial for identifying the precise start and end of each AA episode. This precise timing enables us to incorporate data analysis from both the wristband and video footage into our research. We focus on AI and advanced feature engineering to improve the detection accuracy of AA in people living with dementia. We significantly enhance our chances of detecting AA by using 2 distinct yet cooperative models (one based on physiological data and the other on video analysis). This integrated approach also opens the doors to incorporating additional predictive methods, such as audio-based AA detection. The combined use of video-based systems and wearable wristbands offers deeper insights into AA management. While notable advancements have been achieved, further enhancements are needed, especially in real-time accuracy and system applicability. We are confident that our current research explores a vital area and will contribute new knowledge to the field and lay a solid foundation for future advancements.

## Methods

### Ethical Considerations

The research commenced in 2019. This study was approved by the Joint Research Ethics Board (JREB) at Ontario Shores Centre for Mental Health Sciences and Ontario Tech University (JREB Number: 21-011-B). Informed consent was obtained from all participants’ substitute decision makers, as the participants had advanced dementia and were unable to provide consent themselves. Data collected by the cameras were masked, with any identifiable objects in the video frames blurred (a video demonstration was provided to the research ethics board [REB] chair and privacy officer). All information obtained from participants was kept confidential. Computer-based data were stored in password-protected databases, and paper-based files were kept in locked cabinets. Access to all data were restricted to authorized study personnel, who followed the confidentiality regulations of the JREB. Participants were not compensated for their participation.

This study was approved by the JREB at Ontario Shores Centre for Mental Health Sciences and Ontario Tech University (JREB Number: 21-011-B). Informed consent was obtained from all participants’ substitute decision-makers, as the participants had advanced dementia and were unable to provide consent themselves. Data collected by the cameras were masked, with any identifiable objects in the video frames blurred (a video demonstration was provided to the REB chair and privacy officer). All information obtained from participants was kept confidential. Computer-based data were stored in password-protected databases, and paper-based files were kept in locked cabinets. Access to all data was restricted to authorized study personnel, who followed the confidentiality regulations of the JREB. Participants were not compensated for their participation.

### Study Design

A significant challenge was presented using video cameras to document the behaviors and activities of patients, staff, and visitors in the public spaces of hospital inpatient units. Consent was secured for these patients through substitute decision makers considering their advanced dementia condition. At admission, capacity assessments were performed, and substitute decision makers were approached for permission to involve patients in potential research. The recording of video cameras was restricted to the common areas where patients usually gather during the day, and audio capture was turned off throughout the data collection period.

This study collected participant data using an EmbracePlus wristband [15] and video cameras installed in the GDU and the Geriatric Transitional Unit at the Ontario Shores Center for Mental Health Sciences. A total of 10 participants were recruited based on inclusion criteria, including being aged 60 years or older, a diagnosis of moderate to severe major neurocognitive disorder as determined by the Mini-Mental State Examination [33], and the ability to ambulate independently with or without a walking aid. Additionally, participants had to meet the agitation criteria defined by the Agitation Definition Working Group of the International Psychogeriatric Association [34], with a Functional Assessment Staging Tool scale score between 6a and 6e [35]. Moreover, each unit was equipped with a single AXIS M3077-PLVE Network Camera [36], and one AXIS P3225-VE Mk II camera [37] was installed in the hallway of the GDU to capture relevant footage. Participants wore the EmbracePlus wristband for 24 to 72 hours on 3 separate occasions within a 6-week study period. The wristband collected physiological parameters such as heart rate, electrodermal activity, and skin temperature.

During data collection (Multimedia Appendix 1), clinical staff, who are part of the research team of this study, monitored participants for episodes of AA, noting the start and end times of each event. CCTV cameras recorded footage to provide precise timestamps and additional context, as there can be a delay between observed behavior and recorded notes. To maintain privacy, faces and identifiable features in the video footage were blurred. Once an AA event is identified by the staff in the video, the skeletal points of the recruited participants are extracted and added to the dataset, and all other skeletal points are discarded. The skeletal key points were analyzed using deep learning models to classify episodes of AA. Data from wearable sensors and clinical notes were integrated and analyzed to identify physiological and behavioral patterns preceding episodes of AA. The collected data formed the basis for developing and evaluating our multimodal system for real-time AA detection. The results demonstrate the system’s feasibility and effectiveness in detecting and analyzing AA episodes.

### Event Classification

The proposed system collects the biomarkers using the EmbracePlus wristband, which is considered the state-of-the-art wearable device for continuous health monitoring in the market today [15]. The device combines digital biomarkers, robust sensors, and a user-friendly design to continuously monitor participants with various health conditions. It collects electrodermal data of detected slight changes in skin conductance from the skin surface, and the data of a photoplethysmogram that calculates the pulse rate and pulse rate variability measurements, skin temperature, and raw accelerometry data for motion detection. The collected signals are sent to the cloud-based EmbracePlus Care platform [15] through a Bluetooth-connected gateway (eg, a smartphone).

**Figure 1.**
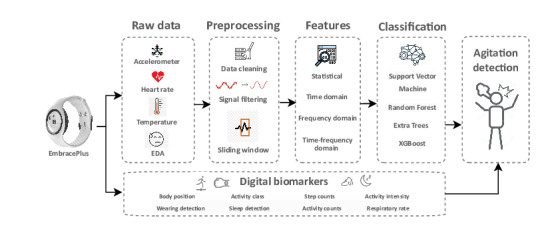
The workflow of the proposed classification system using the EmbracePlus wristband. EDA: electrodermal activity.

The first type of data we deal with is the raw data from the accelerometer, heart rate, temperature, and electrodermal signals. We follow several preprocessing steps to clean, filter, and apply 1-minute window segmentation for the raw signals [21,38]. We then extract features from the signals, as shown in our previous work, from the statistical, time domain, frequency domain, and time-frequency domain with around 150 features [21,38]. Lastly, we evaluate multiple classification techniques, namely Random Forest, Extra Trees, and Gradient Boosting, to classify AA events. The performance of each model is evaluated using standard classification metrics such as accuracy, precision, recall, area under the curve (AUC), and *F*_1_-score. Furthermore, we collect the digital biomarkers that are preprocessed data derived from Empatica’s algorithms and calculate them minute-by-minute. The second type is the digital biomarkers, which include pulse rate variability, respiratory rate, movement intensity, accelerometer magnitude SD, steps, skin conductance level, wearing detection, temperature, and sleep detection as shown in Table 1. Digital biomarkers can effectively and accurately oversee human health from a distance, consistently, and without causing disruption. This applies across a spectrum of health conditions [15]. Figure 1 shows the classification workflow from the EmbracePlus wristband using raw data and digital biomarkers.

**Table 1.** The digital biomarker data description from the EmbracePlus wristband.

Digital biomarkers	Definition
PR^a^	The algorithm uses the photoplethysmogram and accelerometer data for PR monitoring with estimates on 10-second windows.
PRV^b^	The algorithm analyzes the photoplethysmogram for intermittent PRV, using accelerometer signals with nonoverlapping windows.
RR^c^	The algorithm processes the photoplethysmogram and accelerometer data to calculate RR values expressed in breaths per minute.
Movement intensity	The algorithm calculates activity count, steps, and accelerometer SD from the accelerometer sensor.
SCL^d^	SCL estimation from EmbracePlus electrodermal activity signal, output every 1 minute with nonoverlapping windows.
Wearing detection	The algorithm correlates device status with the photoplethysmogram patterns, indicating wearing time.
Temperature	The algorithm analyzes EmbracePlus data for continuous peripheral temperature estimation.

a PR: pulse rate.

b PRV: pulse rate variability.

c RR: respiratory rate.

d SCL: skin conductance level.

The proposed work focuses on investigating the data from people living with dementia using machine learning, 2 of which were thoroughly investigated in our previous work [21,38]. The results concluded the most important features for this problem after performing feature engineering and proved that personalized models on individual patients outperform generic models. In this work, we report the results of the personalized model on 10 different participants from the Ontario Shores Mental Health Institute. We test our system in real-time once we determine the optimal classification system to predict AA events. In the real-time detection phase, real-time raw data is transmitted from the wristband. Following this, features are extracted from each 1-minute window, and these specific features are fed into the customized model to classify whether the data is considered normal or indicative of AA. The outcomes are subsequently transmitted to the backend system, and the health care provider is notified if the patient is agitated.

### Video-Based Analysis

In addition to collecting data on physiological biomarkers, this study incorporates video analysis data for AA prediction. This approach uses an extra cooperative model, which improves our overall AA detection system and allows us to get the precise duration, including start and end times, of the collected AA episodes. Moreover, once an AA episode is detected, the cameras record a previously set preagitation, making it easier to observe any visual preagitation signs. We aim to provide real-time alerts to health care providers for timely intervention. The setup includes 3 CCTV cameras installed and a PC in the attending psychiatric office with access to this footage. Our system operates in 2 phases: the offline phase for manual labeling and model training, and the real-time stage for running the model. To protect the privacy of the participants and the staff present, we blur all faces and run OpenPose (Carnegie Mellon University) to capture the movement data of the participants [39-42]. OpenPose is an advanced real-time system for multiperson 2D pose estimation. It also helps to anonymize individuals in video frames. OpenPose uses convolutional neural networks to detect human body parts and map their skeletal structure onto the image or video frame. This allows for a detailed representation of movement data present in the collected frames. The model is trained on features extracted from skeletal point coordinates instead of the raw video frames. This approach has been recently used by researchers for AA detection in people living with dementia and has proven to be as successful in detecting AA while preserving the privacy of the people present [24,26].

After this, we use a preprocessing phase to enhance the generalizability of the model across various environments and datasets. This phase involves the elimination of extraneous noise that could otherwise impede model performance. The model considers the variations in camera angles and participant positioning within the frame, which can significantly influence the coordinate data. We calculate Euclidean distances and angle measurements between specific skeletal coordinates to determine movements. For example, the measured distance between the torso and feet is useful to identify potential kicking actions, which may indicate AA in certain contexts. Table 2 summarizes all 47 features extracted from these distance and angle measurements. Before training, a feature analysis step is introduced to remove highly correlated features and reduce the dimensionality of the model. This process reduced the features to 39 features. We tested the system on the same 10 participants whose wearable sensor data was used earlier.

**Table 2.** Description of the extracted features from the skeletal data.

Feature	Description
eu_1-eu_14	Euclidean distances between different key point pairs; eu 1 represents the Euclidean distance between key point 1 and the previous position of key point 1
eu_1_3-eu_1_14	Euclidean distances between key point 1 and various other key points
por_2_1-por_14_1	POR^a^ values between key points 2‐14 and key point 1
ang_1_2-ang_1_14	Angles between key point 1 and key points 2‐14

a POR: point of reference.

**Figure 2.**
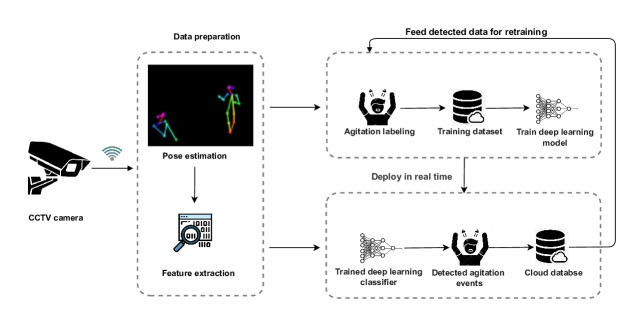
The system architecture of the video-based detection system.

The offline and real-time (online) stages of the system are detailed in Figure 2. In the offline stage, we preprocess and extract features from skeletal data, which are then labeled using nurse notes from patient medical records. Access to the collected videos is restricted to the computer in the psychiatric office, and they are retained only until the AA episodes are accurately labeled with their start and end times. Once the dataset is finalized, all videos are securely discarded. Using this dataset, we train a deep learning model to differentiate between AA and nonagitation events. Our focus is on capturing a range of behaviors, from violent or aggressive actions to repetitive motions such as pacing or chair rocking. Hence, we use models that consider sequences of actions, such as RNN-based neural networks, to effectively recognize these sequences of actions. We specifically use and compare the results of the GRU and LSTM models. Both are adept at analyzing sequences of actions. The LSTM model is designed to capture long-term dependencies within sequences. The sophisticated cell structure of LSTM cells makes it highly effective in maintaining context over long intervals. However, the GRU model uses a simpler architecture that aims to achieve results comparable to LSTM models but with lower computational costs. Both models used in our research are composed of a single LSTM or GRU cell, followed by a fully connected sigmoid layer for the binary classification of AA episodes.

In the real-time stage, we deploy our offline-trained model to classify AA in real-time. The model first processes real-time video data from hospital cameras. Video frames are analyzed using OpenPose, extracting features similar to those in the offline stage. We use a fixed-size window to input frame sequences into the classifier. Upon detecting AA, the system records the event, including a 5-minute buffer before and after the incident to capture the entire context. This approach helps identify potential triggers and patterns that lead to AA. The psychiatrist reviews these detected events for accuracy and confirms AA events, which are then added to our training dataset with appropriate labels. The model is then retrained in the offline stage with the added data. The primary goal of retraining the model is to continuously adapt and improve the model with newly detected data.

## Results

### Overview

A pilot study was conducted to validate the effectiveness and feasibility of the proposed system at Ontario Shores Mental Health Hospital. This initial investigation aimed to provide valuable insights into the system’s functionality, usability, and overall potential before the implementation on a larger population. Details of the enrolled participants and the data collected can be found in Table 3. Upon enrollment, the participants wore the EmbracePlus wristband for 24 to 72 hours. We turned on the cameras installed in the unit during the data collection days to record the participants’ activities. Lastly, we assigned a nurse to observe the participant and provide a detailed report of behavior, AA events, and any abnormal behavior. During the 3 days, we collected 6 AA events, ranging from 2 to 23 minutes per AA event, with a total of 20‐32 minutes of AA labels and 560‐581 minutes of normal labels for each participant. The following sections will present the results from the EmbracePlus wristband and video cameras in detail.

**Table 3.** Overview of the demographic of the participants and the total collected data.

Participant	Gender	Age	Recruitment date	Total collected data (hours)
1	Female	83	August 2023	54.21
2	Female	63	January 2024	48.13
3	Female	77	March 2024	45.3
4	Female	78	March 2024	96.4
5	Female	79	March 2024	99.4
6	Male	79	May 2024	46.7
7	Male	67	August 2024	36
8	Male	68	August 2024	35.5
9	Male	85	September 2024	95.5
10	Male	86	September 2024	54.6

### Performance Evaluation

#### The EmbracePlus Wristband Raw Data

This study uses raw data obtained from 4 wristband signals and personalized models, which achieved superior accuracy in AA detection from people living with dementia in previous research [21,38]. Subsequently, we conducted a comparative analysis of multiple machine learning and deep learning algorithms for AA detection, including Random Forest, Extra Trees, Gradient Boosting, and MLP. We trained and tested a personalized model for every participant and reported the evaluation results in Table 4. The dataset for each participant was randomly split into 70% for training and 30% for testing.

**Table 4.** Comparative performance metrics using raw data.

Participant	Extra Trees			Gradient Boosting			Random Forest			MLP^a^		
Acc^b^	AUC^c^	Recall	Acc	AUC	Recall	Acc	AUC	Recall	Acc	AUC	Recall
1	0.98^d^	0.99^d^	0.99^d^	0.97	0.98	0.98	0.98^d^	0.99^d^	0.99^d^	0.97	0.98	0.98
2	0.90^d^	0.98^d^	0.96	0.88	0.96	0.93	0.88	0.96	0.94	0.88	0.96	0.97^d^
3	0.99^d^	0.99^d^	0.99^d^	0.98	0.99^d^	0.99	0.98	0.99^d^	0.98	0.99^d^	0.99^d^	0.99^d^
4	0.91^d^	0.99^d^	0.97^d^	0.89	0.97	0.96	0.88	0.97	0.99	0.89	0.96	0.98
5	0.99^d^	0.99^d^	0.99^d^	0.98	0.99^d^	0.98	0.98	0.99^d^	0.98	0.95	0.90	0.96
6	0.99^d^	0.99^d^	0.99^d^	0.98	0.99^d^	0.98	0.98	0.99^d^	0.98	0.99^d^	0.99^d^	0.99^d^
7	0.92^d^	0.98^d^	0.97	0.89	0.96	0.96	0.89	0.97	0.96	0.90	0.96	0.99^d^
8	0.91^d^	0.98^d^	0.97	0.91	0.96	0.96	0.88	0.97	0.95	0.89	0.96	0.98
9	0.99^d^	0.99^d^	0.99^d^	0.98	0.99^d^	0.98	0.98	0.99^d^	0.98	0.99^d^	0.99^d^	0.99^d^
10	0.99^d^	0.99^d^	0.99^d^	0.98	0.99^d^	0.99	0.98	0.99^d^	0.98	0.99^d^	0.99^d^	0.99^d^
All	0.85	0.94	0.93	0.98	0.96	0.90	0.93	0.95	0.65	0.99^d^	0.98^d^	0.98^d^

a MLP: multilayer perceptron.

b Acc: accuracy.

c AUC: area under the curve.

d Bold text indicates the best results for each participant in each metric.

The Extra Trees model outperformed the rest of the models for most of the participants, followed by the MLP model, which achieved similar results for 4 of the 10 participants. For example, the Extra Trees model achieved an accuracy of 98.67%, an AUC of 99.1%, and a recall of 99.76% for participant #1. It achieved the highest accuracy and AUC for participant #2 of 90% and 98%, respectively. For participants, #3, #6, #9, and #10, both the Extra Trees and MLP models achieved 99% across all evaluation metrics. These results underscore the efficacy of the chosen features, preprocessing methodologies, and up-sampling techniques. When tested on all 10 participants together, the Extra Trees model achieved a lower accuracy of 85%. The Gradient Boosting and Random Forest achieved higher accuracies in comparison with 98% and 93%, respectively. The Random Forest model, however, performed very poorly in other evaluation matrices, with a recall of 67% for the general model. The MLP model achieved a slightly higher accuracy than Gradient Boosting of 99% and even a higher AUC and recall of 98%, performing very similarly to the personalized models. This highlights the potential for a general model when enough data are collected.

Table 5 presents a summary of the statistical analysis of model performance metrics across all models. As expected, Extra Trees is the top-performing model for participant-level predictions, with the highest mean accuracy of 0.95 and a 95% CI of 0.928-0.986. This indicates consistent performance across the evaluation set. Pairwise statistical tests further confirmed the superior accuracy of Extra Trees compared to Gradient Boosting (*P*=.001), Random Forest (*P*=.006), and MLP (*P*=.002). Although MLP demonstrated a comparable mean accuracy of 0.94 and even outperformed Extra Trees in the general model setting, it exhibited a broader CI (0.909-0.979) and a similar SD (0.04). In terms of other metrics, Extra Trees also achieved the highest mean AUC of 0.98 and a narrow CI of 0.970-0.990. MLP and Gradient Boosting delivered competitive results in AUC 0.97 and recall 0.96. These findings suggest that while MLP performed well in the general model, Extra Trees consistently outperformed other models in the personalized model evaluations. This consistency identifies Extra Trees as the most reliable model for this multimodal system. Additionally, the observed trends highlight the importance of focusing on personalized models to consider individual variations.

**Table 5.** Statistical analysis of model performance metrics across all models.

Metric	Extra Trees	Gradient Boosting	Random Forest	MLP^a^
Mean accuracy	0.95^b^	0.94	0.94	0.94
SD (Acc^c^)	0.04	0.04	0.05	0.04
95% CI (Acc)	0.928-0.986	0.912-0.976	0.905-0.977	0.909-0.979
Paired *P* value(vs Extra Trees)	—^d^	0.001	0.006	0.002
Mean AUC^e^	0.98^b^	0.97	0.96	0.97
SD (AUC)	0.01	0.01	0.01	0.01
95% CI (AUC)	0.970-0.990	0.960-0.980	0.950-0.970	0.960-0.980

a MLP: multilayer perceptron.

b Bold text indicates the best result for each metric.

c Acc: accuracy.

d Not applicable.

e AUC: area under the curve.

Furthermore, we studied the importance of different features for every participant (Multimedia Appendix 2). For participant #1, the top 10 features contributing to accurate AA classification using the Extra Trees model revealed that 5 were from electrodermal activity, 3 from the accelerometer, 1 from heart rate, and 1 from temperature. Notably, the electrodermal tonic mean was the most critical feature, suggesting a strong link between AA episodes and fluctuations in electrodermal activity, which is commonly associated with emotional arousal. For participant #2, the most important features were primarily the accelerometer and temperature signals. Features related to energy, root mean square, and variability in acceleration played a key role in AA classification. Additionally, temperature fluctuations were also significant, suggesting that both movement patterns and body temperature changes could indicate agitation onset in this participant.

For participant #3, the temperature-related features were the most dominant in identifying AA episodes. The maximum temperature value, temperature root mean square, and energy were among the top contributors, highlighting a strong correlation between changes in body temperature and agitation. These findings emphasize the individual variability in AA predictors, reinforcing the necessity of personalized models for accurate classification. While some participants exhibit agitation-related physiological changes in electrodermal activity, others may show significant patterns in movement or temperature variations, underscoring the importance of a multimodal feature selection approach.

**Figure 3.**
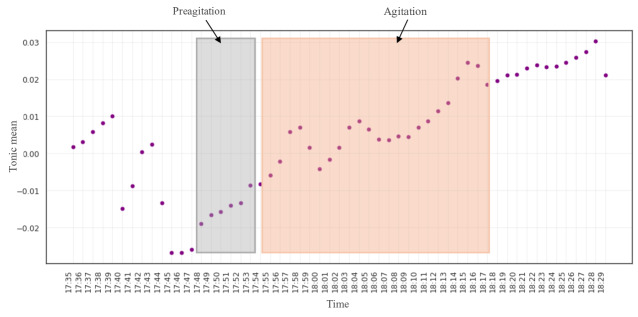
The EmbracePlus wristband raw data with agitation and preagitation annotation: the tonic mean plot for participant #1.

As the electrodermal tonic mean was the top feature to classify AA for participant #1, we investigated the AA labels. Figure 3 shows the tonic mean values of participant #1 from the electrodermal signal during labeled AA events from the camera and nurse notes (highlighted in red). This event occurred during the second day and lasted for 23 minutes from 5:55 PM to 6:17 PM. This observation suggests that the patient’s AA was related to the electrodermal signal connected to the emotions. We also observed an apparent change to the data before the actual AA occurred, which we manually marked as preagitation labels (highlighted in blue).

**Figure 4.**
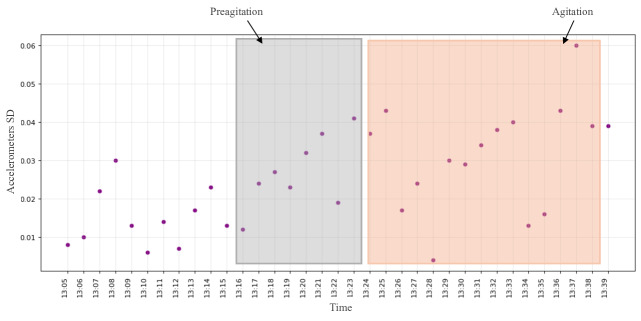
The EmbracePlus wristband raw data with agitation and preagitation annotation: the accelerometer plot for participant #2.

For participant #2, the acceleration features were the top features in identifying the AA event. Figure 4 shows one of the AA events for this participant occurring between 1:24 PM and 1:38 PM using the accelerometer data. Moreover, a change in the pattern of the signal 8 minutes before the event is manually labeled as preagitation.

**Figure 5.**
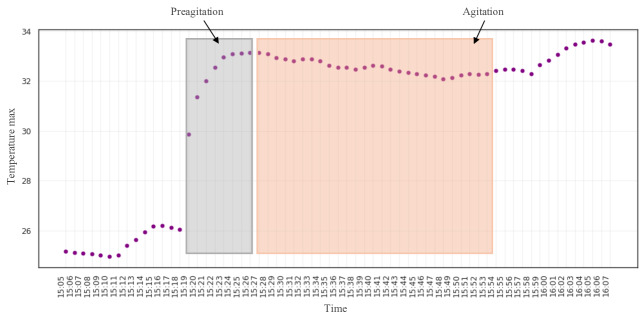
The EmbracePlus wristband raw data with agitation and preagitation annotation: the temperature plot for participant #3. max: maximum.

For participant #3, the most dominant features were the temperature features, so an example of the temperature readings for an AA event is shown in Figure 5. Just as before, a change in the pattern before the observed AA event is manually labeled as preagitation. These observations suggest the potential for detecting preagitation patterns from raw data, enabling the prediction of AA before it occurs.

#### The Wristband EmbracePlus Digital Biomarkers

**Figure 6.**
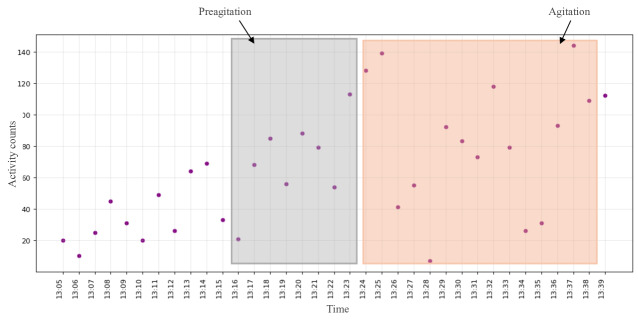
The activity counts from the digital biomarkers data for participant #2.

We explored all the digital biomarkers offered by EmbracePlus. Taking the first 3 participants as an example, we observed that pulse rate, activity counts, and activity class were the leading indicators for AA detection for the first 3 participants. In Figures6^8^ , the same AA events discussed in the previous subsection for the 3 participants are illustrated, and the values during labeled AA events from the camera and nurse notes are highlighted in red. Additionally, we observed a noticeable change in the data before the onset of AA, manually designated as preagitation labels and highlighted in gray. The manual labeling of the preagitation was done after reviewing all the signals for the participants and noting the same change across multiple patterns.

**Figure 7.**
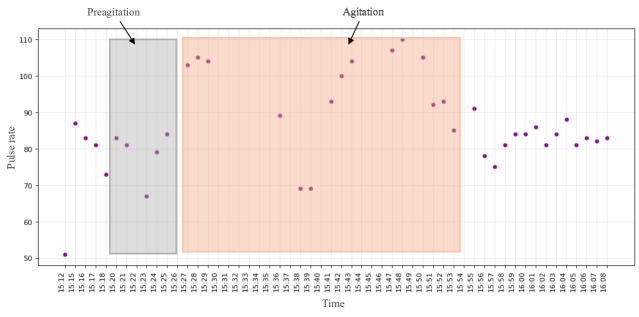
The pulse rate from the digital biomarkers data for participant #3.

**Figure 8.**
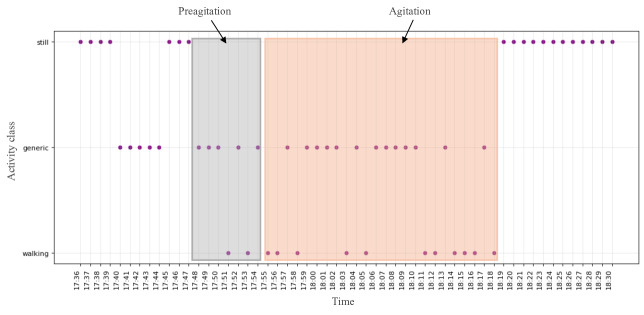
The activity class from the digital biomarkers data for participant #1.

Figure 1 illustrates the activity class for participant #1, extracted from the accelerometer signal, revealing that the participant was in motion rather than stationary during AA and preagitation episodes, indicating body movement during these events. Figure 6 displays the total activity counts for participant #2 from the accelerometer signal. While the normal activity count for the participant ranged between 0‐100 during AA and preagitation events, it surged to 50‐140, signifying heightened activity levels during AA. Finally, Figure 7 presents the pulse rate derived from the heart rate signal for participant #3. Although the participant’s average pulse rate ranged from 55‐80 (SD 12.4) bpm, it increased to 90‐110 (SD 11.6) bpm during AA and preagitation events. Across the raw and digital biomarkers data, we observed that the preagitation occurred from 3:20 PM to 3:27 PM. This indicates that signs of AA behavior occurred approximately 7 minutes before the actual event, suggesting the potential to predict and prevent AA events.

### Performance of Video-Based Detection

We preprocessed our videos using OpenPose and performed feature extraction as described in the methodology section. As AA behaviors are repetitive in nature, we selected RNN models to capture the sequential patterns of these events. Features were extracted from 30-second windows. The window moves 1 second at a time to capture different variations of AA behaviors from the skeletal points. For the classification task, we tested 2 different network structures, which are LSTM and GRU.

In the feature analysis step, we focused on reducing the model’s dimensionality without compromising its performance. Initially, 47 features were extracted based on skeletal movements, including Euclidean distances and angles between key body parts. It is evident from the figures that more features are highly correlated in agitation events than in nonagitation events. This had a strong effect on the feature reduction as only the highly correlated features in both datasets were removed. We investigated the correlation of the features more deeply and tested the model on fewer features based on a correlation threshold. Setting the correlation threshold to 0.8 reduced the number of features to 39 features. The number of features based on the correlation threshold did not change even when the threshold was set to as low as 0.5 due to the huge difference in correlation between both types of events. Both datasets are randomly split into 70% for training and 30% for testing. The training was conducted on a Lambda server equipped with an RTX A6000 GPU, and each model was trained for 100 epochs with a batch size of 256. We used the Adam optimizer to efficiently handle sparse gradients and used a sigmoid activation function for binary classification (AA vs nonagitation). We report the results of all the tests on the testing set in Table 6.

**Table 6.** Video results comparison.

Model	Number of features	Accuracy	AUC^a^	*F*_1_-score	Recall	Time (s)
LSTM^b^	47	0.94	0.98	0.97	0.96	16.1
LSTM	39	0.94	0.98	0.98	0.97	14.7^c^
GRU^d^	47	0.95^e^	0.98^e^	0.98^e^	0.97	30.2
GRU	39	0.95^e^	0.99^e^	0.98^e^	0.98^e^	29.8

a AUC: area under the curve.

b LSTM: long short-term memory.

c Bold text indicates the shortest inference time.

d GRU: gated recurrent unit.

e Bold text indicates the best result for each metric.

We compared metrics such as accuracy, AUC, and *F*_1_-score. We also compared the response time, an essential factor in real-time applications, of all models. As observed in the table, the reduction in the number of features did not affect the performance of the models. The LSTM model, in both cases, achieved an accuracy of 94% and an AUC of 98%. The GRU model reached 95% accuracy and 99% AUC. Although the performance of both models is similar, the response time of the GRU model is double that of the LSTM. The response time for the LSTM was 15.9 seconds when all the features were used in training and was almost one second faster with fewer features. The GRU models, however, had a response time of 30.1 seconds for 47 features and 29.7 seconds for 39 features. The results show that the GRU model is superior across all evaluation metrics, albeit for the response time, where it lags behind the LSTM model by a huge margin. As we aim to detect AA as early as possible, the swifter response time can allow for timely interventions by health care providers in case of any AA event. The high AUC values of both models signify a strong ability to minimize the rate of false positives. This is crucial to ensure the reliability of the model in detecting real AA with lower false alarms, causing less overhead for health care providers. The faster response time posits the LSTM model as possibly the more advantageous model for real-time deployment.

## Discussion

### Principal Findings

The successful implementation of the system within the hospital setting, considering privacy and the positive feedback from patients and health care professionals, highlights the system’s viability in a real-world clinical environment. The system used in this study integrated physiological data from the EmbracePlus wristband and video footage from CCTV cameras, allowing for a comprehensive and multimodal approach to AA detection. The EmbracePlus wristband system demonstrates promising results in detecting and classifying AA and preagitation events in individuals with severe dementia. The AA detection results are reflected in the video detection system, and the preagitation labels can be added to the system from EmbracePlus. The following discussion highlights key findings and their implications, followed by suggestions for future work.

The EmbracePlus wristband, which leverages both raw data and digital biomarkers, demonstrated its efficacy in discerning patterns associated with AA and preagitation. The personalized Extra Trees model emerged as the top-performing algorithm for the raw data, achieving high performance. Furthermore, features such as electrodermal tonic mean, accelerometer activity class, and pulse rate highlighted the significance of identifying AA. Furthermore, the outcomes of our analysis are promising and demonstrate the potential of predicting AA in dementia care settings in real time. We were able to predict preagitation events from all participants at least 6 minutes before the actual AA event. The identification of preagitation patterns in the data suggests that physiological changes precede observable AA behavior. Being the first to explore these patterns in individuals with severe dementia from the EmbracePlus wristband, this study lays the groundwork for a deeper understanding of the dynamics and physiological signatures of AA behaviors. The newfound ability to identify preagitation patterns offers a potential window for early intervention and preventive measures.

Moreover, the video detection system, incorporating CCTV footage and advanced pose estimation techniques, was used along with the EmbracePlus wristband for AA detection. The privacy preservation technique, which follows the REB protocols in the hospital, does not exploit the patient’s personal features or body image without affecting the performance of the proposed model. The LSTM neural network and the GRU networks exhibited robust performances. The high AUC of both models is particularly crucial in the context of health care to minimize the risk of false negatives and ensure that true AA events are accurately identified. During the real-time deployment stage, the model adapts and continuously improves based on the collected data. The labeled preagitation labels collected from the wristband can be fed into the training set of the video detection system to provide insight into detecting preagitation from the video footage. The outcomes of our analysis are promising and demonstrate the potential of both LSTM and GRU neural networks in detecting AA in dementia care settings in real time.

### Future Work

For future work, we will focus on expanding our dataset by recruiting more participants from the hospital. We aim to validate the system over the long term, assessing its stability, generalizability, and adaptability to health care and home care environments. This step is crucial for building a database with a substantial number of AA and preagitation events, which is essential for developing a high-performance detection system using machine learning. Once our detection system is established, we plan to automate the real-time system that is capable of predicting AA. This involves receiving data in real time, classifying the data, and sending notifications to the health care providers if an AA event occurs. Additionally, we plan to improve the real-time AA detection of the video system. We also plan to use the preagitation labels from the EmbracePlus wristband to help our video detection model predict AA before they happen. During the initial stages of this study, health care providers will review and confirm all collected AA events. Their feedback is crucial in refining and enhancing the model’s performance and should aid in identifying any limitations or challenges. Once the model is reliable, it will automatically detect and predict AA with no human intervention.

### Conclusions

This study represents a notable step forward in developing an AA and preagitation detection system for individuals with severe dementia. It uses a comprehensive approach by integrating psychological biomarker sensing and video detection systems. The results demonstrate the feasibility and efficacy of monitoring systems that combine various data sources for AA detection. This study recruited 10 participants from the Ontario Shores Center for Mental Health Sciences Institute. We used the EmbracePlus wristband for continuous health monitoring and video footage from CCTV cameras for real-time observation of AA events. In the preliminary data analysis, the features extracted from the raw data of the EmbracePlus wristband demonstrated exceptional performance in detecting AA events, with the Extra Trees model emerging as the top-performing algorithm for all the personalized models and MLP outperforming the rest of the models for the general model and achieving an accuracy of 98%. Exploring the digital biomarkers further strengthened the system’s classification of AA, preagitation, and normal events. Pulse rate, activity class, and activity counts have emerged as critical indicators for detecting AA. This study revealed the potential for detecting preagitation patterns, showcasing a 6-minute lead time before actual AA events. This early detection capability holds promise for timely intervention and preventive measures. In addition to the EmbracePlus wristband, the video-based detection demonstrated promising results in detecting AA using GRU, achieving a 95% accuracy rate and a robust AUC of 98%. The data analysis’ promising results highlight the potential of the multimodal approach to enhance patient care and safety by predicting AA events. This research will provide new directions for researchers interested in technologies for dementia care and provide challenging propositions in detecting and monitoring, modeling, and evaluating patient-specific interventions for people living with dementia demonstrating NPS.

## Supplementary material

10.2196/68156 Multimedia Appendix 1 Workflow of the data collection process.

10.2196/68156 Multimedia Appendix 2 Feature importance for participants #1, #2, and #3.

## Abbreviations

AA: agitation and aggression
AI: artificial intelligence
AUC: area under the curve
GDU: Geriatric Dementia Unit
GRU: gated recurrent unit
JREB: Joint Research Ethics Board
LSTM: long short-term memory
MLP: multilayer perceptron
NPS: neuropsychiatric symptom
REB: research ethics board
RNN: recurrent neural network
