# Supplementary figures and images for: Multimodal Detection of Agitation in People With Dementia in Clinical Settings: Observational Pilot Study
Source: JMIR Aging. 2025 Jul 15;8:e68156. doi: 10.2196/68156 (PMC12282644; doi:10.2196/68156)

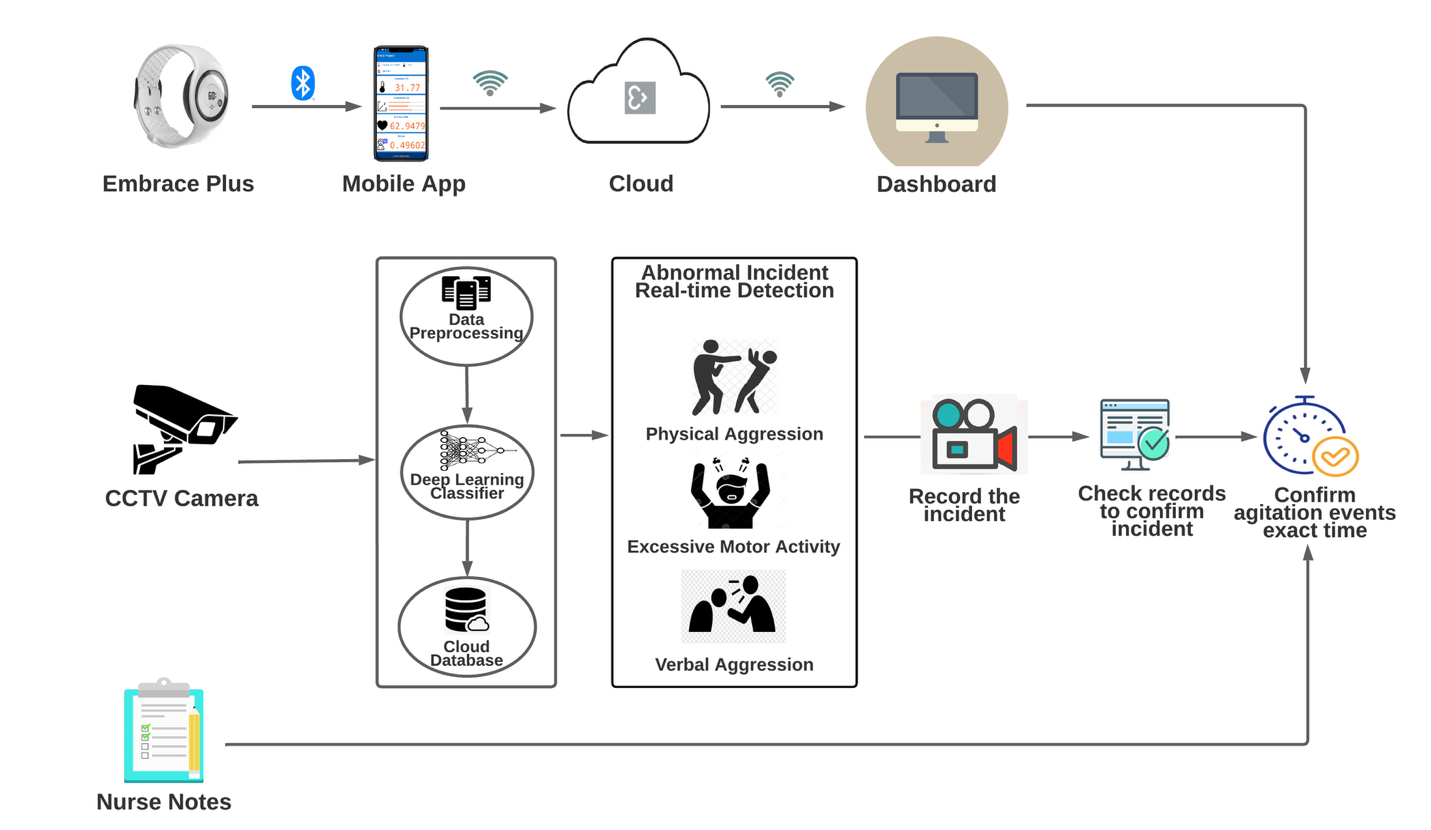

Supplement: Multimedia Appendix 1 [file aging-v8-e68156-s001.docx]

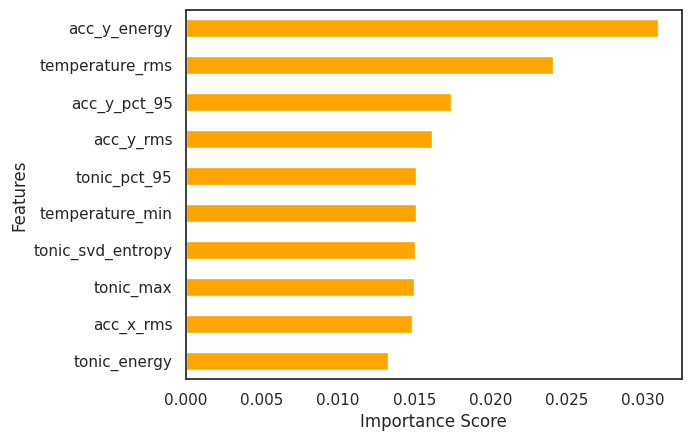

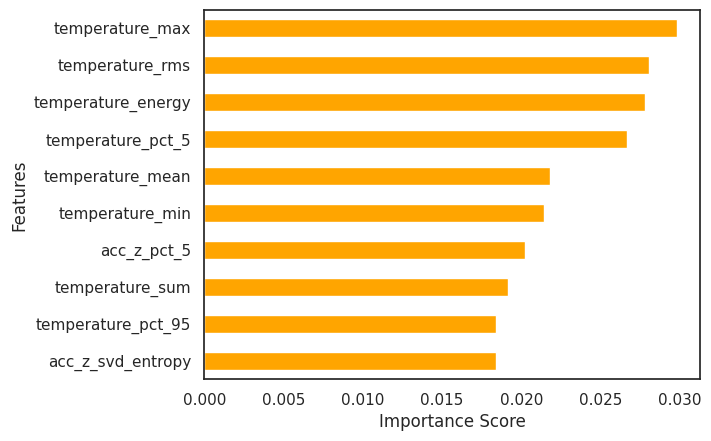

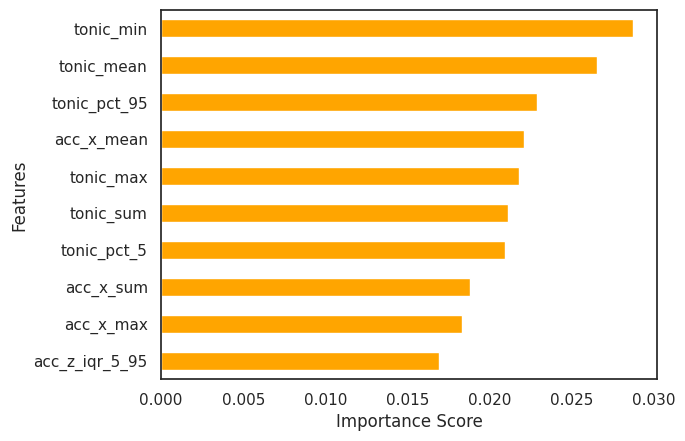

Supplement: Multimedia Appendix 2 [file aging-v8-e68156-s002.docx]
